# DUSPs, to MAP kinases and beyond

**DOI:** 10.1186/2045-3701-2-24

**Published:** 2012-07-09

**Authors:** Ching-Yu Huang, Tse-Hua Tan

**Affiliations:** 1 Immunology Research Center, National Health Research Institutes, Zhunan, Miaoli County, 35053, Taiwan; 2 Department of Pathology & Immunology, Baylor College of Medicine, Houston, TX, 77030, USA

**Keywords:** Phosphatase, DUSP, Signal Transduction, T Cell Development, Immune Regulation

## Abstract

Phosphatases are important regulators of intracellular signaling events, and their functions have been implicated in many biological processes. Dual-specificity phosphatases (DUSPs), whose family currently contains 25 members, are phosphatases that can dephosphorylate both tyrosine and serine/threonine residues of their substrates. The archetypical DUSP, DUSP1/MKP1, was initially discovered to regulate the activities of MAP kinases by dephosphorylating the TXY motif in the kinase domain. However, although DUSPs were discovered more than a decade ago, only in the past few years have their various functions begun to be described. DUSPs can be categorized based on the presence or absence of a MAP kinase-interacting domain into typical DUSPs and atypical DUSPs, respectively. In this review, we discuss the current understanding of how the activities of typical DUSPs are regulated and how typical DUSPs can regulate the functions of their targets. We also summarize recent findings from several *in vivo* DUSP-deficient mouse models that studied the involvement of DUSPs during the development and functioning of T cells. Finally, we discuss briefly the potential roles of DUSPs in the regulation of non-MAP kinase targets, as well as in the modulation of tumorigenesis.

## Review

### The dual-specificity phosphatase family

**Figure 1:**
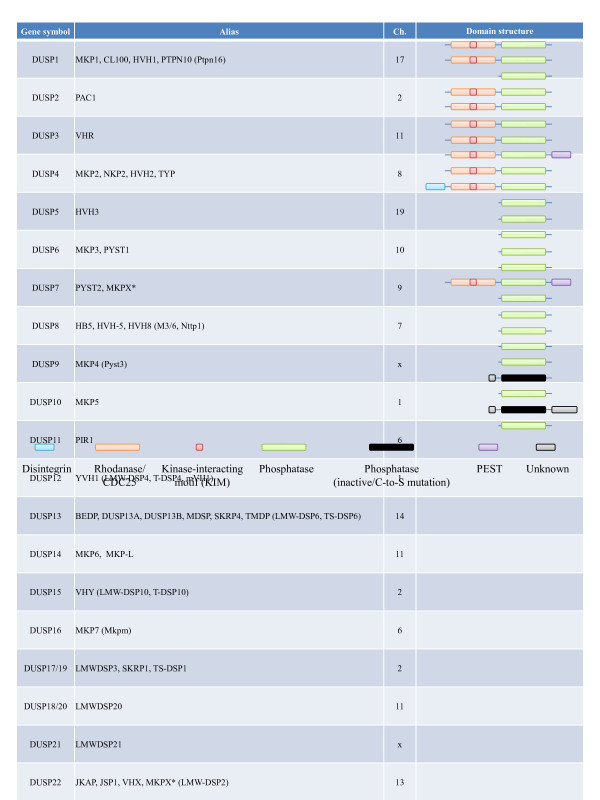
**Summary of DUSP aliases, mouse chromosomal locations, and domain structures.** Gene symbols and selected aliases are from the National Center for Biotechnology Information database via searches for human DUSPs. Selected mouse-specific aliases are shown in parentheses. Chromosome locations (Ch.) and domain structures are manually annotated from the Ensembl database. ^*^, MKPX has been redundantly used for DUSP7 and DUSP22. ^#^, DUSP24 and DUSP26 have been renamed to DUSP26 and DUSP28, respectively.

The dual-specificity phosphatase (DUSP) family proteins are so named for their ability to dephosphorylate both the threonine/serine and tyrosine residues of their substrates. This ability may be attributed to their shallow and flexible enzymatic pockets, which can accommodate both types of phosphorylated residues. Structure-wise, all DUSPs contain a common phosphatase domain with conserved aspartic acid, cysteine, and arginine residues forming the catalytic site. A subset of DUSPs contains an N-terminal region composed of two CDC25 homology 2 domains and an intervening cluster of basic amino acids known as the MAP kinase-binding (MKB) motif or kinase-interacting motif (KIM); this MKB/KIM motif of DUSP interacts with the common domain (CD) of MAP kinases to mediate the enzyme-substrate interaction. Some DUSPs also contain a C-terminal PEST domain or additional N- or C-terminal domains; but the functions of those domains are not well characterized (Figure 1) [1].

**Figure 2:**
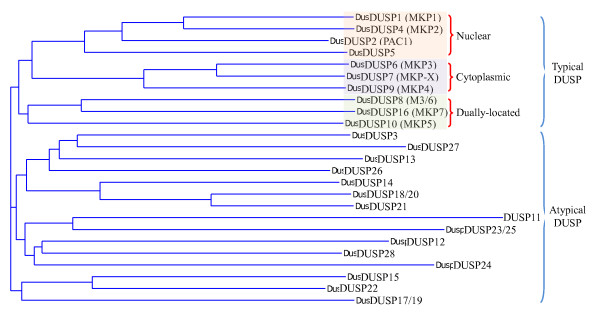
**Alignment of DUSP protein sequences.** Longest variants of mouse DUSPs proteins are from the Ensembl database and are aligned by VectorNTI software. For typical DUSPs, further subgrouping by cellular locations is also shown.

There are currently 25 genes in the Human Genome Organization database designated as DUSPs, namely *DUSP1-28* — with *DUSP17-20*, and −*23* redundantly assigned as *DUSP19-18*, and −*25*, respectively. Within the 25 DUSPs, MS-STYX/DUSP24 and DUSP27 do not contain the conserved cysteine residue for nucleophilic attack (C to S substitution) and thus lack phosphatase activity (Figure 1). These 25 DUSPs can be partitioned, based on their amino acid alignment, into those that contain the MKB/KIM domain and those that do not. DUSPs missing the MKB/KIM domain are generally grouped as atypical DUSPs, while MKB/KIM-containing DUSPs are generally grouped as typical DUSPs or MAP kinase phosphatases (MKPs) (Figure 2). However, there are a few exceptions, with MKP6/DUSP14, JKAP/DUSP22, and MKP8/DUSP26 actually being atypical DUSPs without the KIM domain, and KIM-containing PAC1/DUSP2, HVH3/DUSP5, and HVH-5/DUSP8 not receiving MKP designation (Figure 1). Typical DUSPs can be further divided into three groups based on their predominant subcellular locations (nuclear, cytoplasmic, or dually-located), with this grouping also coinciding with their sequence alignment (Figure 2). Recently, several excellent articles have discussed the potential roles of DUSPs in immune regulation [2-4] and cancer therapy [5-7], and have detailed the regulation of MAP kinases by DUSPs [8,9]. In this review we will focus on the typical DUSPs to discuss the current understandings of their induction and regulation, their immune regulatory roles in peripheral or central lymphoid organ, and their novel regulation of non-MAP kinase substrates.

### Transcriptional, post-transcriptional, and post-translational regulation of DUSPs

The transcription of DUSPs can be activated by factors downstream of MAP kinase [10], such as AP-1. However, the expression of DUSPs is also regulated by other transcription factors. Characterization of the MKP1/DUSP1 promoter found binding sites for Sp1, Sp3, CREB, and USF1 [11]. NF-κB [12] and glucocorticoid receptor [13] could both induce DUSP1 transcription, while HoxA10 was found to induce MKP2/DUSP4 expression [14]. MKP3/DUSP6, in turn, could be induced by ETS-1 following FGF stimulation [15]. More interestingly, p53 was found to regulate the transcription of all four nuclear DUSPs: DUSP1, -2, -4, and −5 [16-19]. In addition to these factors, the transcriptional activation of DUSPs is also regulated by epigenetic modifications [20-22], while DUSP mRNA is subjected to microRNA-mediated gene silencing [23-26]. This complex network for controlling DUSPs’ expression implies diverse functions of DUSPs in the regulation of physiological events following various stimuli, possibly through tissue-, developmental stage-, or activation status-specific induction of DUSPs [27].

As opposed to nuclear DUSPs, which are often highly induced following MAP kinase activation, other typical DUSPs — such as MKP3/DUSP6, PYST2/DUSP7, MKP4/DUSP9, and MKP5/DUSP10 — are constitutively expressed [28]. These DUSPs may serve to define the threshold of MAP kinases activation through preemptive dephosphorylation of MAP kinases. Such a function has been proposed for VHR/DUSP3, and may be coordinated with non-DUSP phosphatases such as PP2A [29].

Protein level of DUSPs is also tightly regulated post-translationally, as the half-lives of many DUSPs are only ~1 h [30-32]. This tight regulation might be partially mediated by the phosphorylation of DUSPs by MAP kinases that inhibit the degradation of DUSPs by proteasomes [31,33-36]. For those DUSPs, their stabilization by MAP kinase-mediated phosphorylation, their short protein half-lives, and sometimes high inducibility suggest that they likely serve as the immediate-early off-switch for MAP kinase signaling.

### Mechanisms of signaling regulation by DUSPs

**Figure 3:**
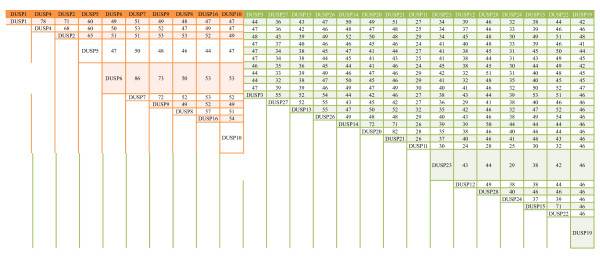
**Conservation matrix of mouse DUSPs.** DUSPs protein sequences are aligned as in Figure 2, and the matrix of conservation is shown. Orange blocks indicate typical DUSPs, while green blocks indicate atypical DUSPs.

DUSPs’ primary mode of action is the dephosphorylation of tyrosine and/or serine/threonine residues and the resulting activity regulation of their substrates. The physiological outcomes of DUSPs’ functions thus hinge on their substrate specificity and phosphatase activity. Unlike kinases, whose substrates are often determined by various well-characterized protein–protein-interacting domains, the substrates for DUSPs may not be similarly defined. For example, while the KIM loosely defines MAP kinase as the substrate for typical DUSPs, atypical DUSPs without the KIM can also efficiently dephosphorylate MAP kinases [29,37]. Furthermore, even with the relatively conserved KIM, the reported specificities for different MAP kinases vary significantly between typical DUSP members (reviewed in [9]), suggesting that the specificity of DUSPs may be refined by regions outside the KIM and phosphatase domains. Indeed, by aligning the longest transcripts for all 25 DUSPs, the results show that amino acid sequence conservation between DUSP members ranges from ~24% to ~86%, with an average conservation of 44%. Within the typical DUSP group, the conservation is on average ~54%, with the lowest at 44% and highest at 86% (Figure 3). Compared with the ~81% conservation within the PP2A/PP4/PP6 family or ~71% conservation within the ERK/JNK/p38 family, this relatively low conservation reflects DUSPs’ variability in the non-phosphatase domains and the inter-domain regions, and may contribute to their broad and divergent substrate specificity.

Between different typical DUSPs, variations also exist in the structural conformation of their phosphatase domains (reviewed in [1]). In these DUSPs, the conserved D, C, and R residues are either well-aligned to form a functional pocket (for DUSP4 [38] and DUSP5 [39]), or are separated in an sub-optimal conformation (for DUSP2 [40] and DUSP6 [41]). In the latter case, substrate-binding to DUSPs can induce conformational changes that significantly increases the phosphatase activity of DUSP2 [42] and DUSP6 [43-45]. Even with a functional pocket, the phosphatase activity of DUSP4 [46] and DUSP5 [35] can still be significantly enhanced by binding with their substrate, ERK. The activity of other DUSPs has also been reported to be enhanced by acteylation [47,48] or phosphorylation [32,49]. These mechanisms likely provide another layer of regulation for maintaining proper phosphatase activity of different DUSPs in multiple signaling pathways.

Regulating the function of DUSP-interacting proteins, however, does not always require DUSPs’ phosphatase activity. It has been shown that DUSPs can modulate the function of MAP kinases by sequestering them in the cytoplasm or nucleus. These sequestrations may serve to retain MAP kinases in the nucleus [50,51], or may prevent them from reaching this destination [52]. Phosphatase-inactive members of the DUSP family — namely DUSP24 and DUSP27 with mutations at the nucleophilic cysteine residue — may thus act through this mechanism to regulate the function of their substrates. As an *in vivo* demonstration of such regulation, over-expression of a phosphatase-inactive and nuclear export-defective mutant of DUSP6 can significantly alter thymocyte maturation [53]. Lastly, since DUSPs and MAP kinase substrates both interact with MAP kinases via MAP kinase’s common CD domain [54], DUSPs may also regulate MAP kinase signaling by competing with MAP kinase substrates for binding with MAP kinases.

### Roles of DUSPs in the regulation of immune cell functions: lessons from *in vivo* studies

In the context of innate and adaptive immune responses, what are the roles of DUSPs in regulating immune cell functions? Such a question is best answered by loss-of-function mutations in genetic studies. In this regard, mice deficient for DUSP1 [55], DUSP2 [56], DUSP4 [57-59], DUSP6 [60,61], DUSP9 [62], and DUSP10 [63] have been reported to exhibit considerably different phenotypes (see also the review article from Salojin and Oravecz [64] for other unpublished mouse lines). Within these mice, DUSP6- and DUSP9-deficient mice did not show any immune-related phenotypes. The dysregulation of immune cell functions in DUSP1-, DUSP2-, and DUSP10-deficient mice has been reviewed previously [3,4]. In summary, DUSP1 was found to be a negative regulator for the production of inflammatory cytokines [55]. DUSP2 was found to positively regulate autoimmune responses in an arthritis animal model [56], while DUSP10 was found to negatively regulate inflammatory cytokine production in innate immune cells but to positively regulate Th1/Th2 cytokine production in CD4 T cells [63]. Several mechanisms may be responsible for the altered cytokine production in DUSP-deficient cells. For example, the altered cytokine productions have been correlated with enhanced activation of MAP kinase and downstream transcription factors such as AP-1 and Elk1 [56,63]. Alternatively, a recent article shows that, in DUSP1-deficient cells, enhanced MAP kinase activation increases AUF1 phosphorylation to maintain the stability of cytokine mRNA [65]. Lastly, DUSPs may also regulate cytokine production and signaling by modulating non-MAP kinase targets, a possibility that will be discussed later in this review.

DUSPs’ regulation of cytokines also provides important insights in the anti-inflammation function of glucocorticoid. Characterization of DUSP1-deficient mice showed that glucocorticoid-mediated induction of DUSP1 and the resulting suppression of JNK and p38 activation contributed to the anti-inflammatory effects of glucocorticoid [66]. A more detailed study of the anti-inflammatory effect of fluticasone suggested that fluticasone induces DUSP1 expression to suppress p38 activation and GATA-3 nuclear translocation, and thereby impairs Th2 cytokine production [67]. However, DUSP2, DUSP4, DUSP9 [68], and DUSP10 [69] have all been found to be induced by glucocorticoid, and are more strongly induced in DUSP1-deficient cells to compensate for DUSP1-deficiency in the induction of anti-inflammatory response [70]. These results suggest that, despite the tissue- and developmental stage-specific expression of different DUSPs, a significant degree of functional overlap and cross-regulation are certainly conceivable within the DUSP family. Similar compensatory up-regulation has also been reported in LPS-induced signaling in DUSP4-deficient mice [71].

DUSP4-deficient mice were recently reported to have impaired inflammatory cytokine production and to be more resistant to LPS-induced septic shock [71]. Interestingly, in a second DUSP4-deficient mouse line, DUSP4 was found to potentiate LPS-induced IL-6, IL-12, TNFα, and prostaglandin E production [58]. Meanwhile, DUSP4-deficiency in these mice resulted in susceptibility to *Leishmania mexicana* infection [58]. Last but not least, results from a third DUSP4-deficient mouse line revealed phenotypes in CD4 T cell proliferation that could be attributed to altered IL-2 response [59]. While the different results from in these independently generated DUSP4-deficient mouse lines may be caused by targeting strategy variations, they may also reflect the complex nature of DUSP transcriptional control, the positive/negative feedback-regulation via MAP kinases, and the compensatory effects between different DUSPs. In this regard, the characterization of *in vivo* functions of DUSPs will need to be performed in well-defined systems, and the results must be interpreted carefully for specific scenarios.

The essential roles of DUSPs in immune regulation are also demonstrated in various reports using other animal models of LPS challenge, bacteria infection, or polymicrobial peritonitis induction. In DUSP1^−/−^ mice, Gram^+^[72], Gram^-^[73,74], and commensal gut bacteria [75] all induce exacerbated inflammatory responses that are associated with increased secretion of inflammatory factors from macrophages or neutrophils. Similar enhanced inflammation has also been observed in DUSP10^−/−^ mice following LPS challenge [76,77]. Interestingly, where live bacteria are used, the enhanced inflammation and supposedly stronger anti-bacterial immune response in DUSP1-deficient mice do not facilitate bacteria clearance, but instead cause a higher mortality rate [73] or increased bacteria burden [74,75]. These outcomes may be attributed to possible bacteria dissemination due to vascular injury [76] or to a novel function of IL-6 that enhances bacteria replication [74]. However, equally significant is the possibility that DUSPs are required for maintaining a balanced immune response by modulating the magnitude and duration of effector functions of immune cells; in other words, DUSPs may be important for fine-tuning immune responses so that these responses become strong enough for keeping pathogens in check, but not too strong to induce excessive tissue damage.

### DUSP-mediated regulation of developing thymocytes

Signals from the MAP kinases are important for thymocyte development and helper T cell polarization (reviewed in [4]). In addition, several DUSPs have been found to be dynamically expressed in differentiating thymocytes [28]. It is thus intriguing that none of the reported DUSP-deficient mice exhibited detectable thymic phenotypes [55-58,60-63]. Our characterization of DUSP4-deficient mice also did not reveal any significant defect in T cell maturation [59]. Nevertheless, two reports utilizing DUSP5 over-expression and DUSP6 dominant-negative mutant, respectively, revealed altered thymocyte differentiation and T cell functions *in vivo*. In the first report, transgenic over-expression of DUSP5 only slightly decreases the number of mature thymocytes and peripheral T cells [78]. However, the DUSP5 transgene increases the signaling threshold of thymocyte selection by decreasing ERK phosphorylation and IL-2 response, which then lead to the selective maturation of autoreactive T cells and subsequently autoimmune skin lesions [78]. In the second report, wild-type bone marrow cells were infected with lentiviral dominant-negative DUSP6 and were then used for reconstitution into congenic recipients; the analyses of donor-originated thymocytes in the bone marrow chimera show that the expression of dominant-negative DUSP6 leads to stronger ERK activation and enhanced thymic positive selection [53]. These results suggest that DUSP5 and DUSP6 may be important for setting the T cell receptor signaling threshold via the modulation of MAP kinase kinetics or activity, so that proper thymic selection can facilitate the production of protective, but not autoreactive or unresponsive, peripheral T cells. Such a hypothesis is also supported by results from the analyses of miR181a-deficient mice, in which DUSP5 and DUSP6, two targets of miR181a, are implicated as the effector molecules for miR181a-mediated regulation in thymocyte maturation [23].

Although the above results clearly demonstrate a potential role for DUSP5 and DUSP6 in the thymus, whether these observed phenotypes reflect the physiological functions of DUSP5 and DUSP6 in T cell differentiation remains to be determined. Specifically, neither of the two independent DUSP6-deficient mouse lines has shown any thymic phenotypes [60,61]. This suggests that different DUSPs may have redundant functions in regulating thymocyte development, so that significant defects can be observed only with transgenic over-expression of wild type DUSPs [78] or dominant-negative mutant DUSPs [53]. It may thus be necessary to cross multiple DUSP-deficient mouse lines to uncover the roles of DUSPs in thymocyte development. In this regard, all typical DUSP genes are located on different chromosomes (Figure 1), making the cross feasible and straightforward. In addition, the generation and characterization of mutant mice carrying multiple defective DUSP alleles should also provide better insights into DUSPs’ other *in vivo* functions.

### Specificity of DUSPs against MAP Kinases: Do *in vivo* or *in vitro* assays tell the true story?

The availability of DUSP-deficient mice also permits the investigation, in a more physiological setting, of DUSPs’ substrate specificity in the context of MAP kinases. In this regard, analyses of DUSP-deficient mice frequently show that the phosphorylation of MAP kinases is not accordingly enhanced based on their previously characterized substrate specificity *in vitro*. For example, the deficiency of DUSP2, a known phosphatase for ERK and p38, does not cause enhanced ERK phosphorylation [56]. Similarly, knockout of DUSP10 does not induce p38 hyperphosphorylation [63]. One may attribute this inconsistency to the use of *in vitro* overexpression system during previous characterizations of DUPS’s substrate specificity, which may not faithfully reflect the outcomes from DUSP-deficient primary cells. Alternatively, the lack of a particular DUSP may be compensated by other DUSPs; due to the large number and broad substrate specificity of DUSPs, this compensatory effect may be more pronounced in DUSP-deficient mice than in mice lacking other signaling molecules. Mechanistically, this compensation may be mediated by other MAP kinase-inducible or constitutively-expressed DUSPs. Finally, it is possible that cross-talks between MAP kinase members [79] also contribute to the observed changes of MAP kinase activation in DUSP-deficient mice. This has been demonstrated in JNK-dependent suppression of ERK activation in DUSP2^−/−^ mice [56], and in DUSP10/16 siRNA-induced, p38-dependent suppression of ERK phosphorylation [80]. All these mechanisms, together with the feed-forward and feed-back modes of DUSP/MAP kinase cross-regulation, will no doubt interfere with the prediction of MAP kinase-related phenotypes in DUSP-deficient mice.

### DUSP-mediated regulation of Non-MAP kinase targets

**Figure 4:**
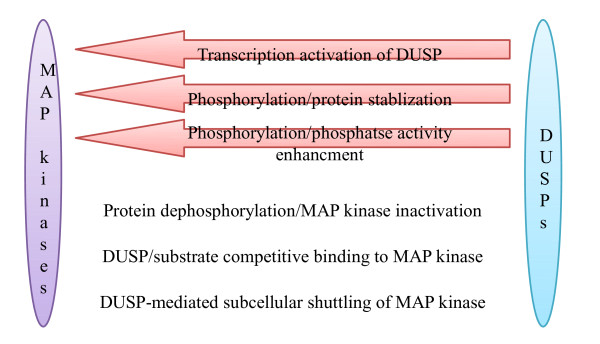
**Mechanisms for the cross-regulations between DUSP and MAP kinase.** Arrows indicate MAP kinase-induced activation of DUSP. Blunted arrows indicate DUSP-mediated suppression of MAP kinase.

Many molecular mechanisms responsible for the cross-regulations between DUSPs and MAP kinases have been discussed (summarized in Figure 4). However, the possibility that typical DUSPs may dephosphorylate non-MAP kinase proteins must also be considered. During our characterization of DUSP4-deficient mice, we found that STAT5 phosphorylation was enhanced in activated DUSP4^−/−^ T cells; physical interaction between DUSP4 and STAT5 was also confirmed in both primary cells and 293T overexpression system. These data thus suggest that DUSP4 may dephosphorylate STAT5 to negatively regulate T cell activation [59]. Similarly, DUSP22 interacts with and dephosphorylates STAT3 after IL-6/LIF treatment in transformed cell lines [81]. Other than STAT family proteins, histone H3 also interacts with and is dephosphorylated by DUSP1 to mediate epigenetic regulation on VEGF-induced gene transcription [82]; FAK co-immunoprecipitates with and is dephosphorylated by DUSP22 at several residues to regulate cell migration in H1299 lung cancer cells [83]. Combined, these results signify DUSP-mediated modifications on non-MAP kinase proteins, an aspect that was somewhat overlooked in the past. Moreover, their effects on STATs also suggest an important role for DUSPs in the regulation of transcription programs downstream of cytokine signaling.

While the above results implicate non-MAP kinase proteins as targets of DUSPs based on both cellular and biochemical analyses, additional reports also make similar claims using RNAi interference or overexpression of DUSPs. In this regard, knockdown of DUSP1 increases angiotensin II- [84], IFNγ- [85], or LPS-induced [86] STAT1 phosphorylation. Similarly, knockdown of DUSP26 regulates neuron differentiation by enhancing PI3K/Akt signaling [87]. However, these results need to be interpreted with caution because the observed effects on non-MAP kinase targets may result from indirect effects. For example, a thorough biochemical study fails to reveal any DUSP1-STAT1 interaction or cross-regulation [88]. Instead, DUSP1 is found to inhibit miR155 expression to induce SOCS-1, thereby attenuating STAT1 activation [89]. Lastly, since DUSPs may regulate cytokine production through MAP kinase-dependent pathways as discussed previously, the observed changes in STAT activation may also be secondary effects due to autocrine or paracrine functions of the altered cytokines. Therefore, although the substrate spectrum of DUSPs may indeed spread beyond MAP kinases, such a conclusion must be sustained by positive results from careful biochemical analyses or *in vitro* functional tests.

## Conclusions and future directions

Compared with kinases, phosphatases have arrived on the stage of signaling regulation nearly a decade late. On the one hand, this recent arrival allows more advanced genetic, structural, and molecular tools to be used, expediting the studies of phosphatase functions. On the other hand, the late entry may also unintentionally limit the breadth and scope of phosphatase-related studies to their functions on kinase regulation. Although more and more reports are focusing on the independent functions of various phosphatases, we believe that the DUSP family proteins deserve more attention for the following reasons. First, the DUSP protein family is a fairly large family with many members. While the theoretical and observed functional redundancy may impede the analyses of their physiological roles, it also provides a safety net if DUSPs are to be targeted by pharmacological reagents in clinical trials. In other words, pharmacological disruption of DUSP functions may not obtain the full-scale effects, as suggested by *in vivo* studies using DUSP-deficient mouse models; however, their effects are likely to be safer, milder, and less dramatic due to the compensatory effects from other DUSPs. In this sense, DUSP inhibitors may behave more like herbal medicines with conceivably fewer side-effects. This concept may provide a different perspective for the development of DUSP inhibitors.

The second reason that DUSPs are a desirable target for medical research is their small size and their simple domain structure, which, to a certain degree, makes the development of small molecule inhibitors less difficult [90]. Indeed, significant efforts have been undertaken by the pharmaceutical industry to develop such inhibitors for medical use (reviewed in [4,91]). With accumulating structural and tissue-expression data, the design and validation of these inhibitors may become more efficient and productive [90,91]. If one can complement the system by generating a comprehensive database of DUSP-deficient mouse models so that off-target effects of these inhibitors can be easily evaluated, we will be one step closer to seeing the applications of DUSP inhibitors in therapeutic treatments.

**Figure 5:**
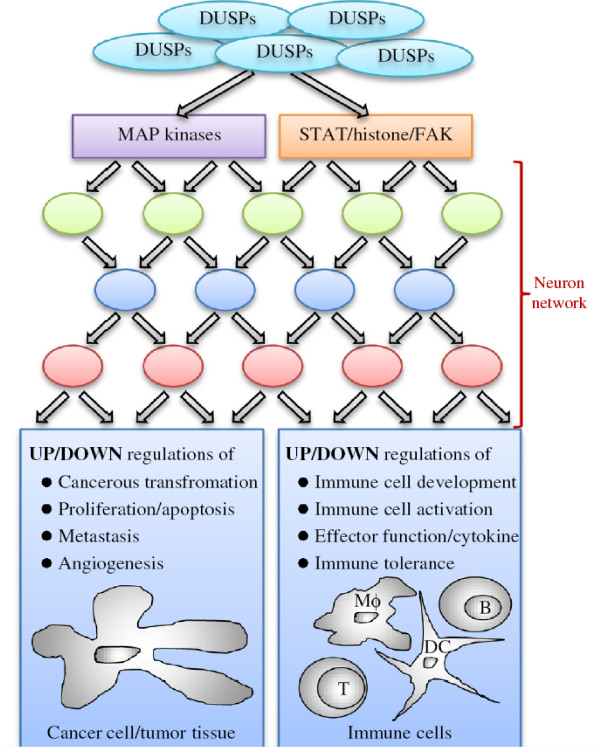
**Schematics of DUSP-mediated regulations in cancerous and immune cells.** Colored circles, unidentified mediators downstream of MAP kinases, STATs, histone, and FAK. DC, dendritic cell. Mϕ, macrophage.

A third point, which has not been emphasized in this review, is the growing list of cancers associated with deregulated DUSP expression (reviewed in [5-7]). These articles detailed how DUSPs may be essential for regulating MAP kinase activities during oncogenic transformation and proliferation/apoptosis of cancer cells. In a separate review, the functions of DUSP-mediated regulations on MAP kinases in tumor metastasis, hypoxia response, and angiogenesis are also discussed [92]. Previous results from our laboratory also demonstrated that DUSPs regulate cancer cell stress response or differentiation via MAP kinase-dependent [93,94] or -independent [87] pathways. In writing this review, we wish to further emphasize the immune regulatory functions of DUSPs, as well as the potential involvement of non-MAP kinase substrates during tumorigenesis. The above regulatory mechanisms are summarized in Figure 5.

Upon glimpsing of Figure 5, one can easily appreciate the “multiple-input, multiple-output” nature of DUSP-mediated modulations on cancer cells. Conceptually, such a scenario is similar to a neuron network in the context of signaling regulations. In such a network, altering one DUSP may cause multiple functional changes of its substrates that are translated into even more diverse physiological functions in the least predictable manner. This argument is partially supported by the fact that the same DUSP is often reported as both overexpressed or suppressed in different tumor cells (reviewed in [91]). In this regard, the design and validation of DUSP inhibitors for treating cancers may be segregated from the investigations of DUSPs’ *in vitro* or *in vivo* functions. While the latter will certainly provide directions for mechanistic studies, they may not assist in predicting the outcome of the treatments. Therefore, we believe that the therapeutic validation of DUSP inhibitors may benefit significantly by expedited migration to *in vivo* models, such as orthotopic tumor transplantations, so that the physiological effects of these treatments can be more faithfully recapitulated.

## Abbreviations

DUSP, Dual-specificity phosphatase; MKB, MAP kinase-binding; KIM, Kinase-interacting motif; CD, Common domain.

## Competing interests

The authors declare that they have no competing interests.

## Authors’ contributions

CYH prepared the initial draft of the paper. THT modified and finalized the paper. Both authors read and approved the final manuscript.
